# Cortical Venous Changes on Susceptibility-Weighted Imaging Predict the Cerebral Collateral Circulation as Confirmed by Digital Subtraction Angiography

**DOI:** 10.3389/fneur.2021.691430

**Published:** 2021-08-27

**Authors:** Yun-Hao Zhan, Yang-Kun Chen, Run-Xiong Li, Gen-Pei Luo, Zhi-Qiang Wu, Yong-Lin Liu, Wei-Min Xiao, Wei-Dong Hu, Cai-Qin Xie

**Affiliations:** ^1^Department of Neurology, Affiliated Dongguan Hospital, Southern Medical University (Dongguan People's Hospital), Dongguan, China; ^2^Department of Radiology, Affiliated Dongguan Hospital, Southern Medical University (Dongguan People's Hospital), Dongguan, China

**Keywords:** acute ischemic stroke, susceptibility weighted imaging, asymmetrical cortical vessel sign, digital subtraction angiography, collateral circulation

## Abstract

**Objective:** Asymmetrical cortical vein sign (ACVS) shown on susceptibility-weighted imaging (SWI) can reflect regional hypoperfusion. We investigated if ACVS could predict the cerebral collateral circulation (CC) as assessed by digital subtraction angiography (DSA) in acute ischemic stroke patients with ipsilateral severe stenosis/occlusion of the anterior circulation.

**Methods:** Clinical data and imaging data of 62 acute ischemic stroke patients with ipsilateral severe stenosis or occlusion of the anterior circulation confirmed by DSA were collected retrospectively. Participants underwent magnetic resonance imaging, including an SWI sequence. ACVS was defined as more and/or larger venous signals in the cerebral cortex of one side of SWI than that in the contralateral side. ACVS was measured using the Alberta Stroke Program Early Computed Tomography score based on SWI. The grading of the cerebral CC was judged using DSA.

**Results:** Of the 62 patients, 30 patients (48.4%) had moderate-to-severe ACVS. According to DSA assessment, 19 patients (30.6%) had a good CC (grade 3–4), and 43 (69.4%) patients had a poor-to-moderate CC (grade 0–2). Among the 30 patients with moderate-to-severe ACVS, only three (10%) patients had a good CC, and 27 (90%) patients had a poor-to-moderate CC; among the 32 patients with none or mild ACVS, 16 (50%) of them had a good CC, and the other 50% had a moderate-to-severe CC. We constructed two logistic regression models with ACVS grading and none or mild ACVS entered into the models, respectively, together with age and large-artery occlusion. In model 1, no ACVS (compared with severe ACVS; OR = 40.329, 95%CI = 2.817–577.422, *P* = 0.006), mild ACVS (compared with severe ACVS; OR = 17.186, 1.735–170.224, 0.015) and large-artery occlusion (OR = 45.645, 4.603–452.592, 0.001) correlated with a good CC. In model 2, none or mild ACVS (OR = 36.848, 95%CI = 5.516–246.171, *P* < 0.001) was significantly associated with a good CC as judged by DSA, adjusted by age and large-artery occlusion.

**Conclusions:** Cortical venous changes in SWI may be a useful indicator for the cerebral CC as confirmed by DSA.

## Introduction

For patients who have suffered acute ischemic stroke (AIS) in the anterior circulation, the key to saving ischemic penumbra is to open the occluded vessels responsible as soon as possible (1). The cerebral collateral circulation (CC) is an essential factor for the early existence of ischemic penumbra (2).

The CC can be divided into three levels. The first level is the circle of Willis. The second level is ophthalmic collateral arteries, pia-mater anastomosis, and other relatively small collateral anastomoses. The third level is neovascularization, which is time-consuming and cannot be evaluated appropriately by digital subtraction angiography (DSA) (3, 4). Commonly used methods for detecting cerebral CC include DSA, multimodal computed tomography angiography (CTA), magnetic resonance perfusion (MRP), and arterial spin labeling (ASL). DSA is the “gold standard” for evaluating a grade-I and -II CC (5). However, it is a traumatic and expensive procedure that necessitates the support of a professional neurointerventional team. Multimodal CTA (6, 7) may be less sensitive for assessment of the leptomeningeal CC compared with that using DSA. DSA and CTA require injection of a contrast agent and carry the risk of contrast-related injuries. MRP (8) or ASL (9) can be used for perfusion assessment rather than direct evaluation for CC. Presence of good CC may enhance the success rate of reperfusion and recanalization in endovascular treatment for AIS (10). CC is a predictor of maintaining brain tissue and good clinical prognosis after AIS, and poorer CC may result in poorer perfusion.

Susceptibility-weighted imaging (SWI) is a magnetic resonance imaging (MRI) sequence. It has high spatial resolution and sensitivity to identify differences in magnetic susceptibility between tissues. Besides its capacity to detect cerebral microbleeds, SWI is considered to reflect oxygen saturation in brain tissues and cerebral vessels. Asymmetrical cortical vein sign (ACVS) denotes more and/or larger venous signals in the cerebral cortex of one side of SWI than that in the contralateral side (11, 12). Several studies show that ACVS is more common in patients with large-vessel occlusion or severe stenosis and reflects cerebral hypoperfusion. Park *et al*. (13) found that total mismatch of diffusion-weighted imaging (DWI)–SWI may be a predictor of good response to treatment in patients with AIS and is associated with cerebral perfusion. In patients with moyamoya disease, ACVS on SWI might be considered as a neuroimaging marker for the evaluation of hemodynamics (14). Moreover, ACVS was observed to be significantly lessened after revascularization (15). ACVS has also been studied in the outcome of AIS. Our recent study (16) found that ACVS might be a useful predictor of early neurological deficits in AIS patients with symptomatic large artery stenosis or occlusion after r-tPA treatment. As perfusion status was significantly affected by CC, it will be interesting to know the relationship between ACVS and CC. To date, very few studies have investigated the relationship between ACVS and CC. Xu *et al*. (17) found that ACVS was associated with good leptomeningeal collateral flow assessed by hyperintense vessel sign (HVS) on T2-FLAIR images rather than DSA. We wished to assess if ACVS could predict the cerebral CC assessed by DSA in AIS patients with ipsilateral severe stenosis/occlusion of the anterior circulation (ISSACS).

## Materials and Methods

### Ethical Approval of the Study Protocol

The requirement for written informed consent was waived by the Ethics Committee of the Affiliated Dongguan Hospital, Southern Medical University (Dongguan, China) because this was a retrospective study.

### Inclusion Criteria

The inclusion criteria were (i) age ≥18 years; (ii) AIS patients with acute infarction of the anterior circulation within 7 days; (iii) complete clinical and imaging data, including, as a minimum, a DWI sequence, SWI sequence, and DSA image: (iv) prestroke modified Rankin scale score ≤ 2.

### Exclusion Criteria

The exclusion criteria were (i) a parenchymatous hemorrhage (PH) evident on CT, (ii) MRI-confirmed AIS of the posterior circulation, (iii) absence of complete clinical and imaging data, (iv) emergency or elective endovascular therapy before MRI completion.

### Participants

We retrospectively recruited 62 patients with AIS in the Affiliated Dongguan Hospital between January 1, 2019, and December 31, 2020. During the study period, 534 patients received DSA, and 188 were diagnosed with AIS by DWI. Of these 188 patients, 126 were excluded because of lack of complete clinical and imaging data (*n* = 65); AIS of the posterior circulation (*n* = 55); diagnosed with Moyamoya disease (*n* = 4); or PH detection by CT (*n* = 2). Eventually, 62 patients formed the study cohort.

### Collection of Clinical Data

We acquired demographic (age, sex) and clinical (National Institutes of Health Stroke Scale score, history of hypertension, previous stroke, diabetes mellitus, tobacco smoking, atrial fibrillation, homocysteine level, hyperlipidemia) data from each patient.

### MRI Assessment

MRI (T1-weighted imaging, T2-weighted imaging, fluid-attenuated inversion recovery, DWI, and SWI) of the brain was undertaken for each participant using a 3.0-T system (Skyra; Siemens Medical, Hamburg, Germany) within 7 days of hospital admission.

The MRI parameters for T1-weighted imaging were time of repetition [TR]/time of echo [TE]/excitation = 1,500/11/1, field of view [FOV] = 220 mm, slice thickness/gap = 4 mm/1.2 mm, matrix = 320 × 320, and time of acquisition = 1 min 26 s. The MRI parameters for T1-weighted imaging were TR/TE/excitation = 4,720/96/2, turbo factor = 15, FOV = 220 mm, slice thickness/gap = 4 mm/1.2 mm, matrix of 512 × 512, and time of acquisition = 1 min 50 s. The MRI parameters for DWI (echo planar imaging (EPI) were TR/TE/excitation = 4,640/67/1, matrix = 192 × 192, FOV = 230 mm, slice thickness/gap = 4 mm/1.2 mm, EPI factor = 91, and acquisition time = 1 min 44 s; three orthogonally applied gradients were applied with b values of 0 and 1000. The MRI parameters for SWI were TR/TE/excitation = 27/20/1, FOV = 220 mm, slice thickness/ gap = 3 mm/0.6 mm, matrix 256 × 256, and time of acquisition = 2 min 28s.

**Figure 1:**
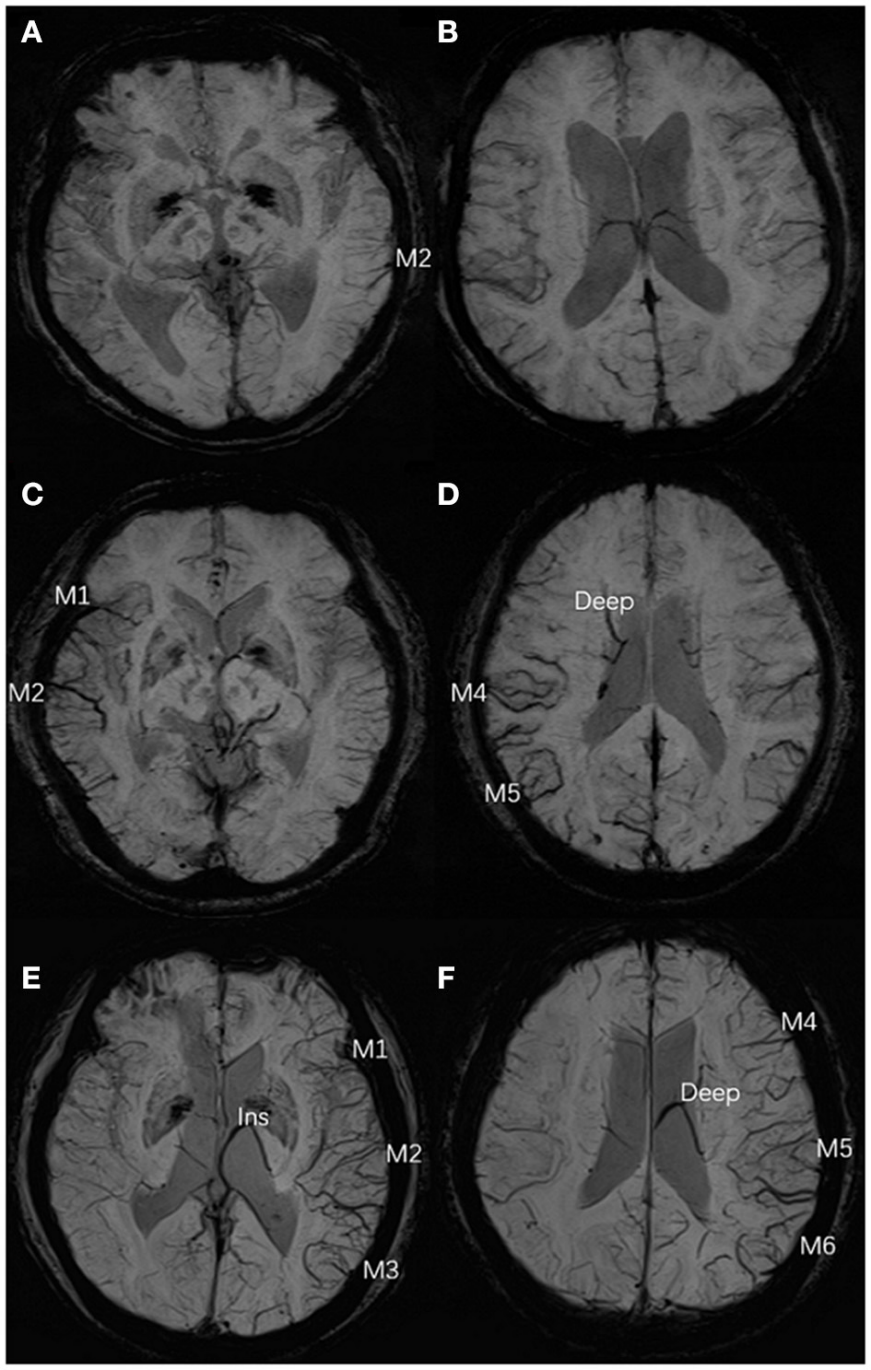
Three typical cases of ACVS, graded as “mild,” “moderate,” and “severe” **(A,B)** In the left hemisphere, ACVS in M2 was evident on SWI and defined as “mild ACVS.” **(C,D)** In the right hemisphere, ACVS in M1 and M2 and M4, M5, and Deep were evident on SWI, and defined as “moderate ACVS.” **(E,F)** In the left hemisphere, ACVS in M1, M2, M3, and Ins and M4, M5, M6, and Deep were evident on SWI and defined as “severe ACVS”. AIS, acute ischemic stroke; ACVS, asymmetric cortical vein sign; M1, anterior MCA cortex; M2, MCA cortex lateral to the insular cortex; M3, posterior MCA cortex; M4, M5, M6, the anterior, lateral and posterior MCA territories immediately superior to M1, M2 and M3, respectively; Deep, deep white matter; Ins, insula; MCA, middle cerebral artery; SWI, susceptibility-weighted imaging.

One experienced neuroradiologist (C.Q.X.) and one trained neurologist (Y.L.L.), blinded to patients' clinical data, assessed the ACVS grade on SWI independently based on the Alberta Stroke Program Early Computed Tomography (ASPECT) score (“Ins,” “Deep,” and “M1-6”) (18). The SWI-ASPECT score ranged from 0 (“no ACVS”) to 8 [“ACVS abutting all middle cerebral artery (MCA) cortical areas”]. The ACVS grade was classified as (1) “none” (no ACVS in any MCA cortical area), (2) “mild” (ACVS in 1–3 defined MCA cortical areas), (3) “moderate” (ACVS in 4–6 defined MCA cortical areas), or (4) “severe” (ACVS in 7–8 defined MCA cortical areas) (19) (Figure 1). Testing of inter-rater and intra-rater reliability of ACVS grade was done in 10 randomized cases. The intra-rater agreement of SWI measurements was good-to-excellent; kappa was 0.83; the kappa for inter-rater agreement was 0.76.

### Evaluation of the Cerebral CC

The decision to undertake DSA was made according to the attending physician. DSA was done using an angiography unit (Allura; Philips Medical Systems, Amsterdam, the Netherlands) after MRI. Carotid and vertebrobasilar angiography were obtained by orthographic projection and lateral projection, and these two projections had a similar angiographic volume and injection rate. Arterial, capillary, and venous phases were imaged to evaluate slow-moving collateral vessels. All patients underwent DSA and MRI during hospitalization.

**Figure 2:**
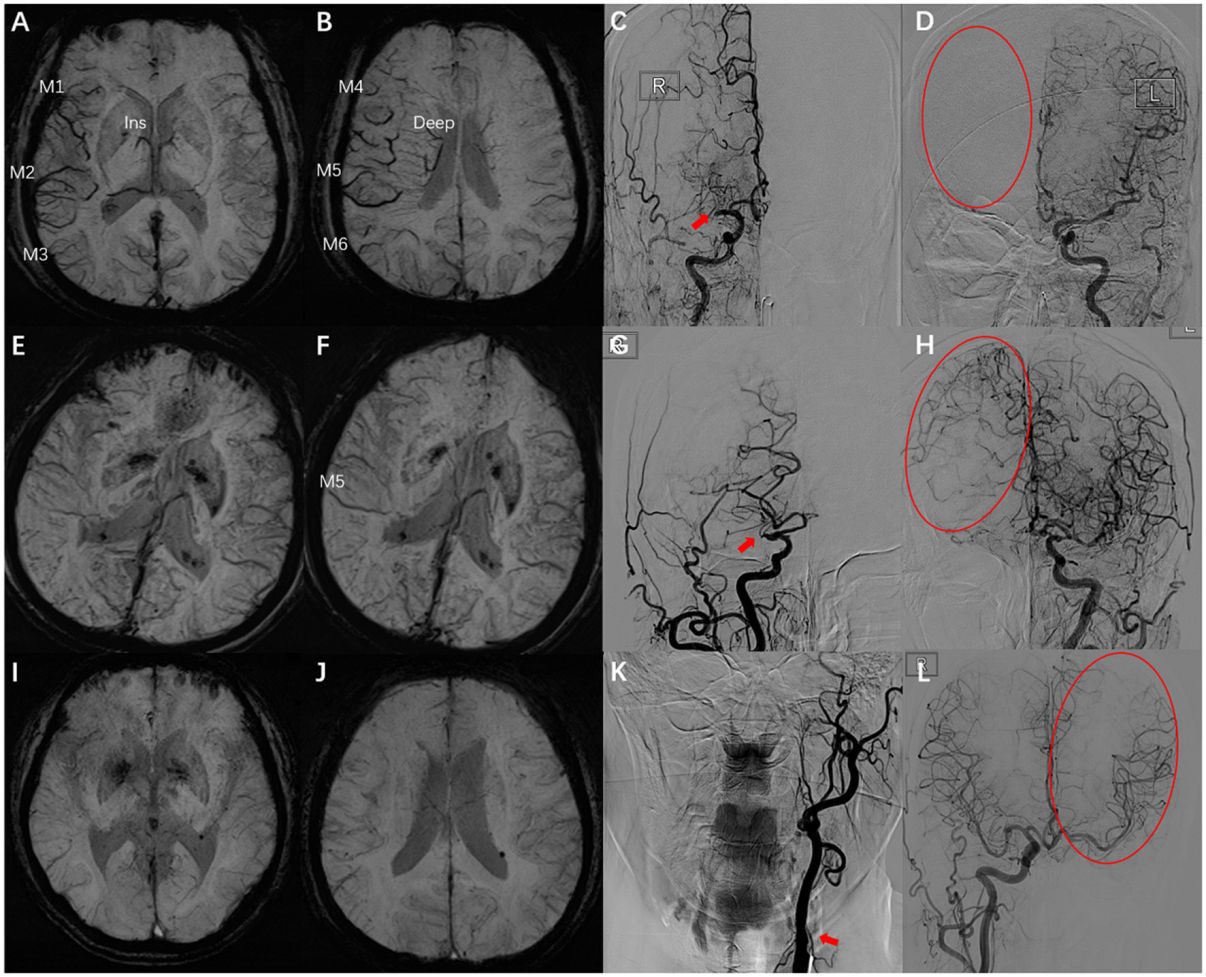
Examples for case selection. **(A,B)** SWI images, ACVS in M1, M2, M3, and Ins and M4, M5, M6, and Deep were evident, ACVS grade was “severe.” **(C,D)** DSA images showed the right MCA was occluded in horizontal segment (red arrow), no visible CC in the ischemic site (red circle), ASITN grade = 0. **(E,F)** SWI images, ACVS in M5 was evident, ACVS grade is “mild.” **(G,H)** DSA images showed the slow CC but complete blood flow to the ischemic bed, ASITN grade = 3. **(I,J)** SWI images, no ACVS in any MCA cortical area, ACVS grade is “none.” **(K,L)** DSA images showed the left ICA was occluded in start section (red arrow), the CC is rapidly and completely perfused to the whole ischemic vascular bed (red circle), ASITN grade = 4. SWI, susceptibility-weighted imaging; ACVS, asymmetric cortical vein sign; Ins, insula; Deep, deep white matter; MCA, middle cerebral artery; DSA, digital subtraction angiography; CC, collateral circulation; ASITN, American Society of Interventional and Therapeutic Neuroradiology; ICA, internal carotid artery; red arrow: responsible vessel; red circle: collateral circulation.

The parameters of DSA images were measured using the measurement tool of Neuosoft Picture Archive and Communication System (PACS)/Radiography Information System (RIS). The criteria for collateral flow map–based collateral grade were chosen based on the American Society of Interventional and Therapeutic Neuroradiology (ASITN) scale (20). ASITN score is the most commonly used to evaluate collateral circulation, which is widely used in the evaluation of collateral circulation on DSA. The latter can be divided into five grades: 0 (no visible CC in the ischemic site), 1 (slow CC around the ischemic site and persistent partial defect), 2 (rapid CC around the ischemic site), 3 (slow CC but complete blood flow to the ischemic bed in late vein), 4 (collateral blood flow can reach the ischemic bed completely and rapidly through retrograde perfusion). Grade 3–4 was designated a “good” CC. Grade 0–2 was designated as a “poor-to-moderate” CC. We chose three cases to describe this grading system (Figure 2). Y.H.Z. and G.P.L. assessed the DSA results independently and were blinded to the SWI results. Twenty cases were selected to test the intra-rater agreement and inter-rater agreement. The intra-rater agreement of DSA measurements was good (kappa = 0.82). The inter-rater agreement of the DSA measurements was good (kappa = 0.74). If the raters disagreed, then a discussion was initiated until consensus was reached.

### Statistical Analyses

Statistical analyses were conducted using SPSS 23.0 (IBM, Armonk, NY, USA). Participants were classified into two groups in terms of a good CC or a not good CC. Continuous variables with a normal distribution are reported as the mean ± SD. Variables with a non-normal distribution are reported as the median and interquartile range. Clinical variables and MRI variables were compared between the two groups using the χ^2^ test, independent-samples *t*-test, Fisher's exact test, or Mann–Whitney *U*-test, as appropriate. Logistic regression analysis was conducted for a good CC confirmed by DSA. The power of ACVS prediction for a good CC was calculated using the area under the curve (AUC) of the receiver operating characteristic (ROC) curve. *P* < 0.05 (two-sided) was considered significant.

## Results

Sixty-two patients (mean age, 59.2 ± 10.6 years; 69.4% male) were evaluated. Thirty-one patients (50%) had occlusion of the internal carotid artery or M1 segment of the MCA. According to the ASITN scale score, 19 patients (30.65%) had a good CC (grade 3–4), and 43 (69.35%) patients had a poor-to-moderate CC (grade 0–2). Among the 30 patients with moderate-to-severe ACVS, only three (10%) patients had a good CC, and 27 (90%) patients had a poor-to-moderate CC; among the 32 patients with none or mild ACVS, 16 (50%) of them had a good CC and the other 50% had a moderate-to-severe CC. Patients with a good CC did not differ significantly from those with a poor-to-moderate CC in terms of age, sex, vascular risk factors, or previous stroke (*P* > 0.05 for all). Patients with a good CC were more likely to have large-artery occlusion (73.7 vs. 39.5%, *P* = 0.013) (Table 1).

**Table 1:** Comparisons of clinical variables and neuroimaging variables between patients with a DSA-confirmed good collateral circulation or not.

	**Whole sample**	**Good collateral circulation**	**Poor-to-moderate collateral circulation**	***P***
	***N*** **= 62**	***N*** **= 19**	***N*** **= 43**	
Age* (years)	59.2 ± 10.6	61.58 ± 7.42	58.12 ± 11.60	0.059
Sex^†^	43(69.4%)	16(84.2%)	37(86.0%)	0.850
Hypertension^†^	53(85.5%)	18(94.7%)	35(81.4%)	0.169
Diabetes mellitus^†^	20(32.3%)	5(26.3%)	15(34.9%)	0.506
Smoker^†^	31(50.0%)	11(57.9%)	20(46.5%)	0.409
Atrial fibrillation^†^	3(4.8%)	1(5.3%)	2(4.7%)	0.918
Previous stroke history^†^	12(19.4%)	4(21.1%)	8(18.6%)	0.822
Hyperlipidemia^†^	31(50.0%)	11(57.9%)	20(46.5%)	0.409
NIHSS score on admission^§^	2(0–15)	3(0–13)	2(1–15)	0.423
**SWI–MRI measures**				
DWI-infarct volume (mm^3^) ^§^	6.5(0.3–115.6)	6.7(1–95.4)	5.8(0.3–115.6)	0.987
MRI timing(days) ^§^	2(0.5–6)	2(0.5–6)	1(0.5–5)	0.024
ACVS grade^†^				0.006
None	20(32.3%)	9(47.4%)	11(25.6%)	
Mild	12(19.4%)	7(36.8%)	5(11.6%)	
Moderate	15(24.2%)	1(5.3%)	14(32.6%)	
Severe	15(24.2%)	2(10.5%)	13(30.2%)	
None or mild ACVS^†^	32(51.6%)	16(84.2%)	16(37.2%)	0.001
DSA timing(days) ^§^	5(0.5–7)	5(0.5–7)	5(0.5–7)	0.691
**DSA measures**				
Occlusion^†^	31(50.0%)	14(73.7%)	17(39.5%)	0.013
ASITN grade^¶^	3(0–6.25)	4(3–4)	0(0–2)	<0.001

*ASITN, American Society of Interventional and Therapeutic Neuroradiology; NIHSS, National Institutes of Health Stroke Scale; SWI, susceptibility weighted imaging; ACVS, asymmetric cortical vein sign; DSA, digital subtraction angiography*.

* *mean (SD), t-test*;

† *n(%), χ^2^ test*;

¶ *n(%), Fisher's exact test*;

§ *median (25Q−75Q), Mann–Whitney U-test*.

We constructed two logistic regression models with ACVS grading and none or mild ACVS entered into the models, respectively, together with age and large-artery occlusion. In model 1, no ACVS (compared with severe ACVS, odds ratio (OR) = 40.329, 95% confidence interval (CI) = 2.817–577.422, *P* = 0.006), mild ACVS (compared with severe ACVS, OR = 17.186, 95%CI = 1.735–170.224, *P* = 0.015), and large-artery occlusion (OR = 45.645, 95%CI = 4.603–452.592, *P* = 0.001) correlated with a good CC. In model 2, none or mild ACVS (OR = 36.848, 95%CI = 5.516–246.171, *P* < 0.001) was significantly associated with a good CC judged by DSA, adjusted by age and large-artery occlusion (Table 2). We also conducted a logistic regression model with poor CC serving as a dependent variable. Moderate-to-severe ACVS did not significantly correlate with poor CC (Supplementary Table 1).

**Table 2:** Logistic regression analysis of a good collateral circulation confirmed by DSA.

	**Univariate logistic regression**			**Multivariate logistic regression**		
**Variables**	**OR**	**95%CI**	***P***	**OR**	**95%CI**	***P***
**Model 1**						
Age	1.035	0.978–1.095	0.236	1.048	0.966–1.136	0.257
DWI-infarct volume	1.000	0.978–1.023	0.987	0.962	0.912–1.016	0.162
MRI timing	1.706	1.043–2.788	0.033	1.536	0.767-3.073	0.225
DSA timing	0.954	0.758–1.201	0.689	0.825	0.534–1.275	0.825
Occlusion	7.407	1.875–29.265	0.004	45.645	4.603–452.592	0.001
ACVS grade			0.019			0.006
Severe	ref			ref		
None	5.318	0.943–29.993	0.058	40.329	2.817–577.422	0.006
Mild	9.100	1.318–59.619	0.021	17.186	1.735–170.224	0.015
Moderate	0.464	0.037–5.749	0.550	0.393	0.030–5.231	0.479
						
**Model 2**						
Age	1.035	0.978–1.095	0.236	1.056	0.975–1.144	0.178
Occlusion	7.407	1.875–29.265	0.004	32.260	4.791–217.233	<0.001
None or mild ACVS	9.000	2.265–35.755	0.002	36.848	5.516–246.171	<0.001

*ACVS, Asymmetric cortical vein sign*.

**Figure 3:**
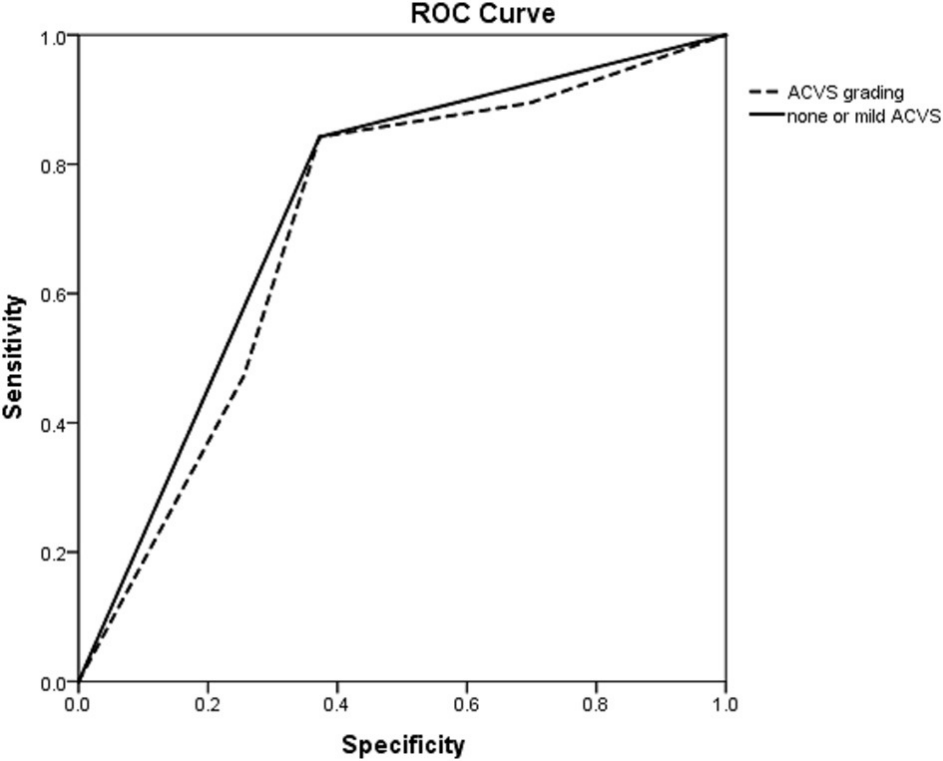
ROC curve of ACVS to predict a good collateral circulation as confirmed by DSA. ROC curve showing the predictive ability of ACVS grading (dotted line) and none or mild ACVS (solid line) for a collateral circulation. ROC, receiver operating characteristic; ACVS, asymmetric cortical vein sign.

The ROC curve showed the predictive ability of ACVS grading and none or mild ACVS for CC. The AUC for predicting a good CC for none or mild ACVS and ACVS grading was 0.735 and 0.706, respectively (Figure 3).

**Figure 4:**
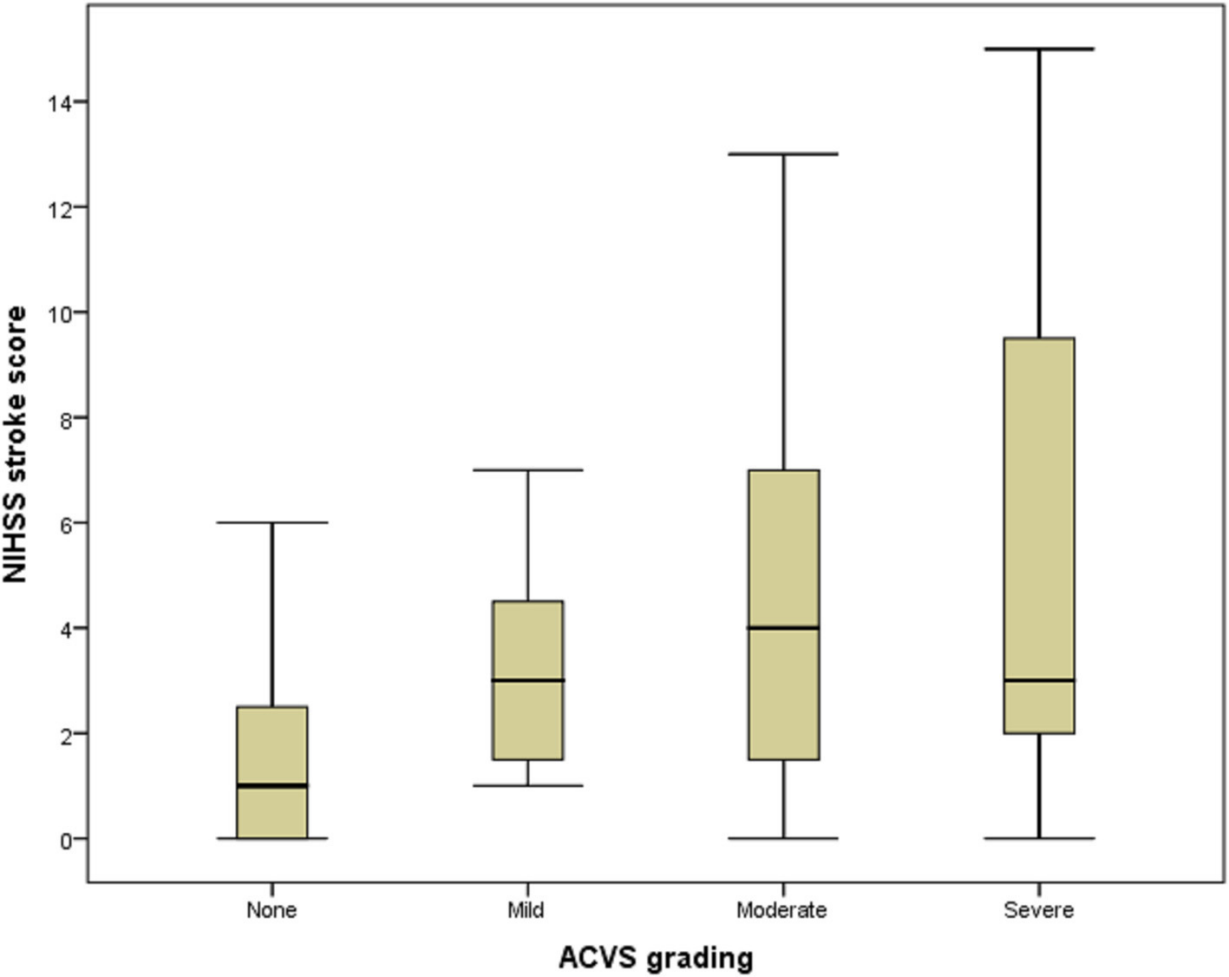
Box plot of the relationship between ACVS and NIHSS stroke score. Box plot showing the relationship between ACVS grading (abscissa) and NIHSS stroke score (ordinate). ACVS, asymmetric cortical vein sign.

A box plot shows the relationship between ACVS grading (abscissa) and NIHSS stroke score (ordinate) (Figure 4). ACVS grading correlated with the severity of stroke (Spearman r = 0.275, *P* = 0.031).

## Discussion

The main finding of our study was that, in AIS patients with ISSACS, ACVS grading (especially none or mild ACVS on SWI) strongly predicted a good CC on DSA. This finding is practical for physicians to acquire, in a prompt and non-traumatic way, CC status using only MRI without the need for contrast-agent injection.

The principle of SWI is dependent mainly on deoxyhemoglobin content in venous blood (21). In patients with AIS, the vasodilation caused by ischemia in the cerebral cortex on the affected side (as well as an imbalance in the supply and demand of oxygen in hypoperfused tissue after cerebral-artery occlusion) leads to a relative increase in the deoxyhemoglobin level in the corresponding draining veins and a relative decrease in the oxyhemoglobin level. This action leads to ACVS, which, as a new imaging marker of CC status, is discussed in a few studies (8, 13). A study on the mismatch between DWI and SWI indicates that the increased oxygen demand of hypoperfused brain tissue leads to an increase in the deoxyhemoglobin:oxyhemoglobin ratio. If the insufficiency of cerebral perfusion can be improved and blood vessels can be recanalized, the risk of irreversible ischemic injury can be reduced (13, 22).

The CC is the basis of ischemic penumbra. A good CC can provide sufficient perfusion for the affected hemisphere (23) and reduce the need of ischemic tissue for a metabolic reserve. Early and rapid identification of ischemic penumbra can help to decide whether intravenous thrombolysis or mechanical thrombectomy is indicated. We found that, in comparison with people with severe ACVS, those without or with only mild ACVS were more likely to have a good CC even if they had symptomatic ISSACS. In addition, none or mild ACVS seems to be a strong indicator for a good CC. These results are in accordance with data from a study by Verma *et al*. (8), which also found that the better the state of the CC, the smaller was the range of ACVS. However, that study had only 33 cases and conducted univariate comparisons only. Determination of ACVS is difficult if bilateral large arteries have severe stenosis/occlusion. Thus, we recruited only AIS patients with ISSACS. Furthermore, we also adjusted these results by age and large-artery occlusion and confirmed this strong association. However, we did not find that severity of ACVS could predict a poor CC. Moderate-to-severe ACVS commonly implicates hypoperfusion, which does not always mean the presence of poor CC. ACVS on MRI-SWI is a non-invasive technique without radiation and contrast allergy risk. Not every AIS patient with large arterial stenosis or occlusion needs a DSA evaluation. It can partially replace DSA assessment of CC in patients with contraindications or unwilling to have DSA for collateral circulation evaluation. However, ACVS is not suitable for patients with bilateral severe large arterial stenosis or occlusion.

In a study of dynamic changes of SWI signals in AIS, Baik et al. (15) found that ACVS was significantly lessened after revascularization. Also, the prognosis of these patients was, in general, not poor but, for patients with hypoperfusion who did not have brain improvement in a timely and complete manner, ACVS on SWI indicated that these patients would be more prone to early neurological deterioration. Previously, we also found that ACVS can predict early neurological deterioration effectively in AIS patients with symptomatic large-artery stenosis/occlusion after treatment with recombinant tissue plasminogen activator (24).

ACVS was difficult to quantify accurately. We used the ASPECT scoring to evaluate the range of ACVS. ASPECT scoring was initially used for the measures of the early CT ischemic changes range in predicting benefit with intravenous thrombolysis. A lower ASPECT score implicates a larger range of involvement. ACVS reflects the change of vein shape and number and is difficult to quantify. SWI-ASPECT scoring can be used to evaluate the range of ACVS as recommended by some studies. Only one study (25) attempted to define ACVS quantitatively using susceptibility percentage change. However, to the best of our knowledge, no studies have successfully quantified the extent of ACVS using objective methods.

The strengths of our study were that the CC was confirmed using DSA (the gold standard) and that we adjusted for confounders in our analyses. However, our study had four main limitations. First, the sample size of our study was small, and the regression model might have been not very stable. Second, we recruited only AIS patients with ipsilateral arterial lesions. Thus, this finding may be valuable only for unilateral severe stenosis/occlusion in the anterior circulation and not applicable for bilateral lesions. Third, as mentioned before, ACVS is difficult to quantify, and we assessed it *via* a semi-quantitative method. Last, we did not have dynamic evaluation of ACVS as no patients in our stroke center would have SWI examination before thrombolysis or thrombectomy according to the guideline of our stroke center.

## Conclusions

Our study provides evidence for the reliability of ACVS in judging the CC in treatment of AIS in the anterior circulation. Further longitudinal studies using larger patient cohorts and more objective ACVS quantification, e.g., using machine-learning methods, are warranted to justify this finding.

## Data Availability Statement

The raw data supporting the conclusions of this article will be made available by the authors, without undue reservation.

## Ethics Statement

The studies involving human participants were reviewed and approved by Ethics Committee of Affiliated Dongguan Hospital, Southern Medical University. The ethics committee waived the requirement of written informed consent for participation.

## Author Contributions

Y-HZ and Y-KC conceptualized and designed the study, acquired and analyzed data, and drafted the manuscript for intellectual content. Y-HZ and G-PL took measurements. Y-LL and C-QX measured SWI data. R-XL, Z-QW, W-MX, and W-DH selected and recruited the patients. All authors contributed to the article and approved the submitted version.

## Conflict of Interest

The authors declare that the research was conducted in the absence of any commercial or financial relationships that could be construed as a potential conflict of interest.

## Publisher's Note

All claims expressed in this article are solely those of the authors and do not necessarily represent those of their affiliated organizations, or those of the publisher, the editors and the reviewers. Any product that may be evaluated in this article, or claim that may be made by its manufacturer, is not guaranteed or endorsed by the publisher.
