# Supplementary material for: Cortical Venous Changes on Susceptibility-Weighted Imaging Predict the Cerebral Collateral Circulation as Confirmed by Digital Subtraction Angiography
Source: Front Neurol. 2021 Aug 27;12:691430. doi: 10.3389/fneur.2021.691430 (PMC8430322; doi:10.3389/fneur.2021.691430)
Supplement: Supplementary file 1 [file Table_1.DOCX]

**Supplement Table 1. Logistic regression analysis of a poor collateral circulation confirmed by DSA**

| Variables | OR | 95%CI | *P* |
| --- | --- | --- | --- |
| Age | 0.959 | 0.906–1.015 | 0.149 |
| Occlusion | 0.137 | 0.039–0.483 | 0.003 |
| Moderate-to-severe ACVS | 2.720 | 0.817–9.054 | 0.103 |

**ACVS, Asymmetric cortical vein sign**
